# Can Online Discussion Sites Generate Quality Data for Research Purposes?

**DOI:** 10.3389/fpubh.2017.00156

**Published:** 2017-07-06

**Authors:** Helen Smith, Alpaslan Bulbul, Christina J. Jones

**Affiliations:** ^1^Lee Kong Chian School of Medicine, Nanyang Technological University, Singapore, Singapore; ^2^Brighton and Sussex Medical School, Brighton, United Kingdom

**Keywords:** social media, health information seeking, qualitative research, internet, online

## Introduction

We have recently presented orally the preliminary findings of some research where we derived our data from online discussion sites. Our research described parents’ and caregivers’ experiences of administering medications to young children (1, 2). Different aspects of these data have been presented at four different conferences (each in a different country, and across two continents). On each occasion, our audience of clinicians and researchers have been divided about the credibility and quality of our results. In response to these divergent opinions encountered, we have reflected on whether online discussion sites can contribute to quality research in the arena of health.

Our own foray into the analysis of the content of online discussion sites was unplanned; it was precipitated by a delay in the approval and recruitment processes for a conventional qualitative research study. Our original plan was to use one-to-one interviews with the parents of young atopic children to explore their experiences of administering health-care treatments. The atopic child may suffer from eczema, asthma, rhinoconjunctivitis, and food allergy and therefore may require many different treatments. The formulations of these treatments are diverse, including inhalers, nasal sprays, liquid medicines, eye drops, and topical preparations. It was the complexity of some children’s treatment regimens that stimulated our interest in understanding how parents coped with sustained, complex treatment administration.

For our analysis of online data, we first identified websites where parents discuss the care of their children. We then searched on these, using such terms as “how to eye drops toddler,” substituting in turn other medication delivery methods for the term “eye drops.”

Data were plentiful; for example, the transcripts of online discussions relating to the administration of eye drops extended to 39 pages. Caregivers described in detail their own experiences and responded to requests for help with pragmatic suggestions. Presentation was sometimes informal, with errors of literacy and spelling but the message was always clear. A thematic analysis (3) identified three major themes: children’s negative reactions to having eye drops, physical restraint techniques adopted by parents to administer eye drops, facilitators and bribes used to ensure adherence. The accounts we identified were very powerful, engaging, and alerted us to previously unrecognized challenges and the parental distress these difficulties generated. We fully recognized that this approach had limitations and was not a substitute for our original research plan, but it did form a useful adjunct. First, analysis of online discussion lacked the specificity that we were seeking; for example, the discussions relating to eye drops included any ophthalmological conditions, not just the atopic eye conditions of interest to us. Second, this research method did not allow us to explore the impact of administering multiple medications, but rather focused on individual medications. Nonetheless, it has provided us with novel data that will impact on clinical practice, and it has informed protocol refinement for our definitive face-to-face interviews with the parents of atopic children.

Our audiences recognized the richness of the data, particularly when it raised issues encountered by parents, that they, as health-care professionals or clinical scientists, were previously unaware. However, the critics in our audiences challenged our data on issues of its quality; they queried its truthfulness, highlighted our lack of knowledge of our respondents, and were concerned about bias and ethics. Below we take some of the questions posed of our data and discuss to what extent they are justified or can be rebutted.

## Are Parents Telling us the Truth?

We acknowledge that our analysis was of data generated by a group of people who had not been convened for research purposes, it was what is sometimes referred to as “naturally occurring” (4) or “non-reactive” data (5). Nonetheless, this does not automatically threaten the quality of the data.

Some of our audiences felt that the descriptors of medication administration were so vivid that they may have been written to create maximum impact and generate empathy, using terms such as “tall stories” or “drama queen accounts” to describe some parental posts. Others questioned the veracity of some accounts, could giving eye drops to a toddler result in a mother being “*really frazzled*” and going to bed in tears, or a father “…*currently in the shed after the last episode, where she* [the toddler] *got so upset; he was holding her and she got so stressed*.”

Why might we as researchers be confident the data are true? We are distant from the generation of the data, and as passive observers we have had no opportunity to probe or to ask follow-up questions. However, qualitative data sources have traditionally included written materials, such as letters and diaries, and the integrity of these data is rarely questioned. Like contributions to online discussion, these documents were written spontaneously, not in response to a research question and without awareness that the writing would subsequently be subjected to analysis. It is also important to recognize that online interactions, such as email, have previously been reported to result in more honest responses, especially from those who are asked to reveal sensitive, personal information (6). Our data focused around parents expressing their own inadequacies of an aspect of childcare, so one can imagine anonymity and participating in the protected and familiar environment of one’s own home could facilitate, not deter, honesty.

## Who Exactly are in your Sample?

This concern is partially shared by us as health service researchers. We recognize our inability to describe the characteristics of our participants; we will never be able to generate for a journal manuscript a table of our participants, describing gender, age, socioeconomic status, education achievement, or occupation. However, we can assume that they all have experience caring for a toddler, as they were engaging in a thematic discussion on this topic. In addition, there is generic information available about the different parenting networks we accessed, for example, the purpose of the website and number of visitors each month. Therefore, it is possible to compile a “case study” of each contributing discussion site to enable the reader to understand more about the context in which these data were generated. Griffiths et al. have developed a useful framework for characterizing networks that could be utilized to generate a fuller description of a site and its activities (7).

## How Inclusive is this Method of Data Collection?

In qualitative research, one is not seeking to enumerate experiences or to achieve representativeness, but rather explore the range and breadth of experience. Online discussion by definition excludes those people without Internet access. Year on year the percentage of users has increased, such that in 2017, 99% of 16- to 34-year olds had Internet access (8). However, there remain differences with respect to age (only 41% of the over 75s access the Internet), gender (less females than males), fewer disabled people, and those who are socioeconomically disadvantaged. Therefore, online discussions may differentially exclude the experiences of some sectors of society. In contrast, the convenience of the Internet may enable the voices of some commonly excluded people to participate in research, for example, those with mobility problems, parents of young families, or those in living in rural settings. Some have debated that for online discussion boards and forums, people respond at their convenience instead of waiting for a turn (as in verbal interactions) that could provide an opportunity for more reserved participants to contribute (9).

## Surely, these Data are Biased?

This could be so, but it may be argued that since the researcher is not leading on data collection and is “invisible” to the participants, this may reduce response bias. The questions, conversation, and information discussed arise spontaneously and naturally, eliminating the potential for the researcher to influence the discussion. Similarly, the risk of responders feeling their responses must be skewed to produce answers that are socially desirable is greatly reduced. In the analytical phase, there remains the need for the researcher to be attentive to their own bias, and if appropriate to voice their prejudices and assumption when presenting their interpretation of results.

## Should such Analyses of Online Discussion be Allowed?

Before embarking on any research project, it is an essential to consider whether ethical approval and consent of participants are required. In the context of qualitative analysis of first person narratives from the Internet, the answer is often, but not universally, no. When describing the justification for this, Katherine Morton Morrison used the analogy of “traditional cork-and-paper bulletin boards” (10). She argued that if one posts information on a board in a private office, to which only limited people have a key, then privacy can be assumed, but when posting information in a public place, such as a corridor, one relinquishes the expectation for privacy, and disseminates the data to a wider audience. Nonetheless, it is essential to respect the privacy of discussants, by removing any potential identifiers and paraphrasing to ensure that from the data presented no individual can be identified. When a site is password protected then consent and ethics need to be discussed and addressed fully. The British Psychological Society’s Ethic Guidelines for Internet-mediated Research (2013) addresses in detail the distinction of between public and private domains and implications for scientific value and potential harm, helping the researcher make decisions within the context of a particular piece of research (5).

## Judging Quality of Data

What is quality data? What parents reported made sense, they described situations one could envisage arising. A series of questions for monitoring the quality of the data derived from online discussions have been suggested (10) and incorporating the answers to these within one’s presentations and manuscripts may help diffuse challenges from an audience and reviewers who skeptical about the quality of data generated from online discussion sites:

- Do these data make sense when compared with data collected by other means? If not, do these data represent negative cases?
- Where on the Internet were these data found?
- Does the site were these data found encourage postings from only one perspective?
- Was the material posted in response to a posted comment? What were these comments?
- Are postings to the site submitted to some review procedure, or are the items simply posted at will?

## Other Ways Online Discussion Sites can Generate Research Data

The discussion above has focused on just one form of Internet-mediated research, qualitative analysis of the content of online discussion posted on social networks. Such analyses are mostly cross-sectional [for example, a study of breastfeeding in public (11), types of support needed by patients with systemic lupus erythematosus (12)], but there is also the potential to do a longitudinal analysis looking for trends over time. Content analysis is the frequently used methods to analyze the text, but more recently, conversation analysis has been applied to better capture the interactive phenomena (13).

There are also ways in which data can be remotely acquisitioned from the Internet using researcher-created surveys. Lewis et al. invited parents and soon-to-be parents of Mumsnet to complete the survey on their views and likely uptake of non-invasive testing for trisomy 21 (14). Their response rate was 25%, which is good for a community survey, and because participants completed a survey it was possible to ascertain their demographic characteristics, although the characteristics of the non-responders remained unknown. The rate of participation can be very fast; another survey on Mumsnet of the views of women on induction of labor at term for women over 35 generated 663 responses within 24 hours, exceeding the 500 preplanned target (15).

## Conclusion

Our presentation of an analysis of online discussions appeared to generate a schism between those who saw the potential of this “naturally occurring data” and those who defended more traditional ways of data collection to understand the patients’ and carers’ experiences. We propose that online discussion sites can provide an extremely valuable source of rich data for research purposes if the researcher is attentive to existing ways of achieving methodological rigor and applies the normal principles of ethical research. People’s narratives on the Internet can have an important role in understanding health-related issues. Interaction through digital social networks can lead to the identification of patient problems that health professionals may not have encountered or realized the enormity of.

## Author Contributions

AB conducted the research under the guidance of HS and CJ. The commentary was prepared by HS and CJ.

## Conflict of Interest Statement

The authors declare that the research was conducted in the absence of any commercial or financial relationships that could be construed as a potential conflict of interest.

## References

[1] Bulbul A Jones CJ Mukhopadhyay S Smith HE Parents’ experiences of administering asthma medications to their children – thematic analysis of online forum discussions and blog entries. Allergy (2015) 70:639–40.

[2] Bulbul A Jones CJ Mukhopadhyay S Smith HE Experiences of parents administering eye drops to their children-thematic analysis of online blog entries and forum discussions. Allergy (2015) 70:211.

[3] Braun V Clarke V Using thematic analysis in psychology. Qual Res Psychol (2006) 3(2):77–101. 10.1191/1478088706qp063oa

[4] Potter J Hepburn A Qualitative interviews in psychology: problems and possibilities. Qual Res Psychol (2005) 2:281–307. 10.1191/1478088705qp045oa

[5] British Psychological Society. Ethics Guidelines for Internet-Mediated Research. INF206/1.2013. Leicester: Author (2013). Available from: www.bps.org.uk/publications/policy-and-guidelines/research-guidelines-policy-documents/research-guidelines-poli

[6] Murray CD Sixsmith J E-mail: a qualitative research medium for interviewing? Int J Soc Res Methodol (1998) 1(2):102–21. 10.1080/13645579.1998.10846867

[7] Griffiths F Dobermann T Cave JA Thorogood M Johnson S Salamatian K The Impact of Online Social Networks on Health and Health Systems: a scoping review and case studies. Policy Internet (2015) 7:473–96. 10.1002/poi3.97 27134699 PMC4841174

[8] Office of National Statistics. Statistical Bulletin: Internet Users in the UK: 2017. London: ONS (2017). Available from: https://www.ons.gov.uk/businessindustryandtrade/itandinternetindustry/bulletins/internetusers/2017

[9] Hiltz SR Wellman B Asynchronous learning networks as a virtual classroom. Commun ACM (1997) 40(9):44–9. 10.1145/260750.260764

[10] Robinson K. Unsolicited narratives from the internet: a rich source of qualitative data. Qual Health Res (2001) 11(5):706–14. 10.1177/104973201129119398 11554197

[11] Boyer K. Affect, corporeality and the limits of belonging: breastfeeding in public in the contemporary UK. Health Place (2012) 18:552–60. 10.1016/j.healthplace.2012.01.010 22366299

[12] Mazzoni D Cicognani E Sharing experiences and social support requests in an internet forum for patients with systemic lupus erythematosus. J Health Psychol (1014) 19:698–696. 10.1177/1359105313477674 23479300

[13] Giles DC. Observing real-world groups in the virtual field: the analysis of online discussion. Br J Soc Psychol (2016) 55(3):484–98. 10.1111/bjso.12139 26916617

[14] Lewis C Hill M Silcock C Daley R Chitty LS Non-invasive prenatal testing for trisomy 21: a cross-sectional survey of survey users’ views and likely uptake. BJOG (2014) 121(5):582–94. 10.1111/1471-0528.12579 24433394

[15] Walker KF Bugg GJ Macpherson M Thorton J Induction of labour at term for women over 35 years old: a survey of the views of women and obstericians. Eur J Obstet Gynaecol Reprod Biol (2012) 162:144–8. 10.1016/j.ejogrb.2012.02.016 22424584

